# The development of an outcome measure for liaison mental health services

**DOI:** 10.1192/bjb.2017.28

**Published:** 2018-06

**Authors:** Else Guthrie, Mathew Harrison, Richard Brown, Rajdeep Sandhu, Peter Trigwell, Seri Abraham, Shazada Nawaz, Peter Kelsall, Rachel Thomasson

**Affiliations:** 1 University of Leeds, UK; 2 Leeds and York Partnership National Health Service Foundation Trust, UK; 3 Manchester Academic Health Sciences Centre, UK; 4 Pennine Care National Health Service Foundation Trust, UK; 5 Lancashire Care National Health Service Foundation Trust, UK; 6 Salford Royal National Health Service Foundation Trust, UK

## Abstract

**Aims and method:**

To develop and pilot a clinician-rated outcome scale to evaluate symptomatic outcomes in liaison psychiatry services. Three hundred and sixty patient contacts with 207 separate individuals were rated using six subscales (mood, psychosis, cognition, substance misuse, mind–body problems and behavioural disturbance) plus two additional items (side-effects of medication and capacity to consent for medical treatment). Each item was rated on a five-point scale from 0 to 5 (nil, mild, moderate, severe and very severe).

**Results:**

The liaison outcome measure was acceptable and easy to use. All subscales showed acceptable interrater reliability, with the exception of the mind–body subscale. Overall, the measure appears to show stability and sensitivity to change.

**Clinical implications:**

The measure provides a useful and robust way to determine symptomatic change in a liaison mental health setting, although the mind–body subscale requires modification.

**Declaration of interest:**

None.

Routine outcome measurement is important in mental health settings and can facilitate improvements in quality and outcome.^1^ At present, there is no recognised, specific, routine outcome measure for liaison psychiatry services. The Health of the Nation Outcome Scales (HoNOS) have been used routinely in general psychiatry settings for the past 20 years,^2^ but attempts to adapt them for liaison settings have never been realised. Liaison mental health services operate in a variety of different settings and treat people with a wide range of complex and heterogeneous clinical problems, which makes the development of a single outcome tool challenging.^3^

The Faculty of Liaison Psychiatry of the Royal College of Psychiatrists has developed an outcome framework (FROM-LP),^4^ which includes measures of patient and referrer satisfaction, and a generic clinician-rated measure, the Clinical Global Impression Improvement Scale (CGI-I).^5^ This measure, however, is not specific for liaison settings and does not generate individual symptom ratings.

The aim of this project was to develop and pilot a liaison outcome instrument to be used for local service evaluation, to supplement the FROM-LP framework and to provide data on symptom outcomes. Our intention was to develop a brief, acceptable and easy to use measure that covered common liaison mental health problems, with good reliability, stability and sensitivity to change. This paper describes the development of the measure and preliminary results from its use in two local acute hospital services in Manchester.

## Method

Items included in the measure were determined using a modified version of the mini-Delphi method.^6^ We ran three cycles of the mini-Delphi process using panels of clinicians (nurses and psychiatrists) working in different types of liaison mental health services in the North West of England, including acute hospital wards, emergency departments, out-patient and community liaison services, and liaison services for older adults. A pool of potential items (64 items) was initially generated, and a prototypic measure was produced which then underwent 6 months of field testing in a Manchester liaison mental health service. Following this, the measure underwent a series of modifications, including the exclusion and rewording of many items. A subsequent 12-month testing period generated further minor revisions.

The final measure was piloted in this study. It had 22 items consisting of six subscales (mood, psychosis, cognition, substance misuse, mind–body problems and behavioural disturbance) and two additional items (side-effects of medication and capacity to consent to medical treatment). Each item was rated on a five-point scale from 0 to 5 (nil, mild, moderate, severe and very severe). In response to feedback from clinicians, a contextual subscale was also added to represent items which may not necessarily change because of a liaison intervention but may influence or affect outcome (e.g. prior history of severe mental illness). These items are meant to be rated at baseline only (Table 1).

**Table 1:** Liaison outcome measure subscales and items

Subscale	Items
Mood	1. Low mood
	2. Suicidal ideation or self-harm
	3. Psychological adjustment to physical illness
Psychosis	4. Perceptual disturbances
	5. Abnormal thought content
	6. Abnormal mood (excluding depression)
Cognition	7. Problems with orientation
	8. Problems with concentration
	9. Problems with memory
Substance misuse	10. Alcohol-related problems
	11. Illicit drug-related problems
	12. Proprietary medication problems
	13. Acute alcohol or drug withdrawal
Mind–body	14. Disproportionate disability
	15. Excessive or major worry about physical health
	16. Pain
	17. Disproportionate treatment-seeking behaviour
Disturbed behaviour	18. Agitation or aggressive behaviour
	19. Non-adherence to treatment
	20. Consciousness and hypoactivity
Additional items	21. Side-effects of psychotropic medication
	22. Problems with capacity to consent to medical or surgical treatment
Contextual items	Physical illness
	Physical disability
	Intellectual difficulties
	Psychosocial stressors
	Enduring mental health problems
	Social function
	Activities of daily living

### Settings

Both services involved in the evaluation were based in Greater Manchester. The first was a consultant-led ward-based service for working adults, which operates on a 09.00 to 17.00 h basis from Monday to Friday, based in a hospital with 850 beds. The second was a consultant-led liaison service for older adults, which operates from 09.00 to 17.00 h, Monday to Friday, based in a hospital with 778 beds. The older adult liaison service sees patients on general hospital wards and has a broad reach within the community, including residential and nursing homes, intermediate care units, hospices, home visits and an out-patient clinic in a local mental health unit for older people.

### Acceptability and ease of use

Acceptability was measured by asking clinicians who used the measure to record the time taken to complete the measure, and to rate on a seven-point Likert scale the ease or difficulty of completion (1 = very easy, 4 = neither easy nor difficult, 7 = very difficult). Feedback was obtained from nine clinicians, including one consultant liaison psychiatrist, one consultant liaison older adult psychiatrist, three higher trainees in psychiatry, one specialist liaison nurse, two core psychiatry trainees and one FY2 trainee.

### Interrater reliability

Interrater reliability was assessed by independent paired raters. Paired ratings were obtained when a patient was assessed on the same day by different members of the same team, or when jointly assessed by a trainee and a senior colleague for the purposes of a workplace-based assessment. Ratings were made separately, without consultation between the raters. Agreement between raters was assessed using intraclass correlation coefficients (ICC).

### Sensitivity to change

The sensitivity to change of an instrument is its ability to accurately detect changes if they occur. For the purposes of this evaluation, change was determined by the Clinical Global Outcome Scale-I,^5^ which is recommended by FROM-LP^4^ and was completed routinely as part of clinical care for patients who were reviewed on at least two occasions. The CGI-I is a seven-point scale (1 = very much improved, 2 = much improved, 3 = minimally improved, 4 = no change, 5 = minimally worse, 6 = much worse, 7 = very much worse).^5^ Patients were divided into three groups: improved (a score of 1 or 2 on the CGI-I), no change (a score of 3, 4 or 5 on the CGI-I) and deteriorated (a score of 6 or 7 on the CGI-I).

It was hypothesised that, if the liaison measure was sensitive to change, there would be a significant difference between the above three outcome groups, and the improved group would show significant improvement on pre and post ratings of the liaison measure, while the deteriorated group would show significant worsening on the liaison symptomatic scores, and the no-change group would show no difference on pre and post scores. The effect size^7^ (*M*_2_ − *M*_1_/s.d._1_, where *M*_2_ = mean at time 2, *M*_1_ = mean at time 1, s.d._1_ = s.d. at time 1) for each group was also calculated.

### Comparability

It was beyond the scope of this service evaluation project to compare all of the subscales of the measure with appropriate recognised, validated instruments. However, it was possible to compare two of the subscales of the liaison instrument with recognised, validated measures that are used routinely in the Manchester liaison services. The CORE-10^8^ is a brief outcome measure comprising ten items, which has been widely adopted in the evaluation of counselling and psychological therapies in the UK. The CORE-10 is recommended by FROM-LP for appropriate subgroups of patients. The Confusion Assessment Method (CAM)^9^ is a standardised evidence-based tool that enables clinicians to identify and recognise delirium quickly and accurately in both clinical and research settings.

It was hypothesised that the CORE-10 scores would correlate highly with the depression subscale of the liaison measure, but not with the other subscales. As the CAM scale produces a positive or negative outcome, patients who scored positively on the CAM were compared with those who had a negative score (i.e. no evidence of confusion). It was hypothesised that those who had a positive score on the CAM would score significantly higher on the cognitive subscale of the liaison measure than those who had a negative score (i.e. no delirium).

This project was checked using the Health Research Authority website to determine whether or not it would be classed as research, and discussed with the local Research and Development lead. There was collective consensus that it should be classed as a local service evaluation.

### Statistical methods

Data were collated and stored, and descriptive statistics were completed using SPSS version 22. Further statistical analysis used the R statistical programming language (version 3.2.5) with the assistance of the ‘RKWard’ graphical user interface (https://rkward.kde.org/), as well as the ‘irr’ (https://cran.r-project.org/web/packages/irr/irr.pdf) and ‘psych’ (https://cran.r-project.org/web/packages/psych/psych.pdf). packages. Normally distributed data were compared using either independent or paired-sample *t*-tests (for before and after comparisons). Non-parametric tests were used for comparison of data that were not normally distributed.

## Results

A total of 360 patient contacts with 207 separate individuals were rated using the liaison outcome measure. One person had two separate episodes of care under the liaison team, resulting in 208 individual episodes of care. One hundred and thirteen people had only one rating, 64 people had two ratings, 18 people had three ratings, eight people had four ratings, two people had five ratings, and one person each had six, seven and ten ratings, respectively.

There were 45 parallel assessments of the same individual at the same point in time. Of these, 41 were paired ratings and four involved three raters. There were 78 pre–post ratings which were of the same individual at different points in time. Of these, 47 were undertaken by the same rater and 31 by a different assessor. Demographic information was recorded for 198 individuals, of whom 104 (52.5%) were male. The mean age was 52.6 years (s.d. = 21.7 years).

### Scale acceptability

The ease of use of the scale was rated for 228 (63.3%) contacts (*x* = 2.1; s.d. = 1.1). The time taken to complete the measure was recorded for 233 (64.7%) contacts (*x* = 2 min, 30 s; s.d. = 2 min, 8 s).

### Interrater reliability

Table 2 shows the ICC for each item of the scale as rated by 45 rater pairs. Kappa (Κ) scores for 15 of the 22 items of the scale and five of the seven contextual factors demonstrated ‘good’ (Κ = 0.61–0.80) or ‘very good’ interrater reliability (Κ = 0.81–1.00), using agreement categories as described by Landis and Koch.^10^ Four items involving the mind–body subscale showed very low kappa scores (14, 15, 16 and 17).

**Table 2:** Intraclass correlation coefficients (ICC) for items of the liaison outcome measure

Measure item		*n*	ICC (95% CI)
1	Low mood	36	0.827 (0.687–0.908)***
2	Suicidal ideation or self-harm	41	0.802 (0.658–0.889)***
3	Problems with psychological adjustment to physical illness	34	0.656 (0.413–0.812)***
4	Perceptual disturbances	40	0.929 (0.869–0.962)***
5	Abnormal thought content	42	0.920 (0.856–0.956)***
6	Abnormal mood (excluding depression)	38	0.828 (0.693–0.906)***
7	Problems with orientation	41	0.861 (0.754–0.923)***
8	Problems with concentration	33	0.816 (0.660–0.905)***
9	Problems with memory	31	0.821 (0.662–0.910)***
10	Alcohol-related problems	39	0.825 (0.691–0.904)***
11	Illicit drug-related problems	32	0.921 (0.844–0.960)***
12	Proprietary medication problems	37	0.947 (0.899–0.972)***
13	Acute alcohol or drug withdrawal	40	0.954 (0.915–0.975)***
14	Disproportionate disability	36	0.224 (−0.109–0.511)
15	Excessive or major worry about physical health	38	−0.0523 (−0.362–0.268)
16	Pain	37	0.299 (−0.023–0.565)*
17	Disproportionate treatment-seeking behaviour	37	0.211 (−0.117–0.498)
18	Agitation or aggressive behaviour	42	0.776 (0.620–0.873)***
19	Non-adherence to treatment	41	0.518 (0.253–0.710)**
20	Consciousness and hypoactivity	42	0.805 (0.665–0.890)***
21	Side-effects of psychotropic medication	34	0.546 (0.259–0.744)**
22	Problems with capacity to give informed consent to treatment	31	0.593 (0.307–0.780)**
1–22	Scale total	45	0.799 (0.662–0.889)***
**Subscale scores**			
A	Mood	45	0.768 (0.614–0.865)***
B	Psychosis	45	0.924 (0.866–0.958)***
C	Cognition	45	0.802 (0.667–0.886)***
D	Substance misuse	45	0.930 (0.876–0.961)***
E	Mind–body	45	0.253 (−0.041–0.506)
F	Behaviour	45	0.748 (0.584–0.853)***

*n*: number of rater pairs.

*P < 0.05; **P < 0.001; ***P < 0.0001.

The ICCs and their 95% confidence intervals for the contextual items were as follows: physical health problems (*n* = 43; ICC = 0.496; CI = 0.233–0.692**); physical disability (*n* = 37; ICC = 0.601; CI = 0.347–0.772***); intellectual difficulties (*n* = 35; ICC = 0.670; CI = 0.437–0.819**); psychosocial stressors (*n* = 35; ICC = 0.696; CI = 0.476–0.843***); enduring mental health problems (*n* = 19; ICC = 0.750; CI = 0.459–0.896***); social function (*n* = 28; ICC = 0.556; CI = 0.237–0.767**); and activities of daily living (*n* = 28; ICC = 0.727; CI = 0.491–0.864***).

With the exception of the mind–body subscale, all subscales of the measure showed ‘good’ or ‘very good’ interrater agreement (Table 2). Agreement for the total score was ‘good’ at 0.799. This increased to ‘very good’ with an ICC of 0.845 (CI = 0.734–0.911, *P* < 0.001) when the mind–body subscale was excluded from the total score.

### Sensitivity to change

Seventy-eight patients had a baseline assessment and a final rating, at least 1 week apart. There was an overall improvement on the liaison outcome measure, with a baseline mean of 15.68 (s.d. 10.90) and a post-intervention mean of 8.41 (s.d. 7.66). This was statistically significant (*t* = 5.28, d.f. = 77, *P* < 0.001). Thirty-seven of these patients were classed as showing improvement on the CGI-I (a rating of much improved or very much improved), 35 patients were classed as showing no change (a rating of minimally improved, no change or minimally worse) and five patients were classed as showing a deterioration (much worse or very much worse). One rating for the CGI-I was not recorded, so this individual could not be classified. Table 3 shows the mean scores for each of the three outcome groups, at the baseline assessment and the final assessment. The change in outcome among the three groups was also significantly different (Kruskal–Wallis test, *P* < 0.001).

**Table 3:** Baseline and post-intervention scores, change scores and effect sizes for patients in the Clinical Global Impression Improvement Scale (CGI-I) improved, no change and worse groups

CGI-I outcome category	Baseline		Post-intervention		Liaison change score		*P*-value (pre–post)	Effect size
	Mean	s.d.	Mean	s.d.	Mean	s.d.		
Improved (*N* = 37)	20.00	12.04	4.54	5.16	17.61	11.65	<0.001	1.29
No change (*N* = 35)	12.77	8.08	11.91	8.03	0.68	7.86	0.477	0.08
Worse (*N* = 5)	4.80	5.36	12.80	8.38	−8.75	5.73	0.027	−1.64

### Comparability with the CORE-10

Twenty-three patients completed the CORE-10. For these patients, there was a significant correlation between the mood subscale and the CORE-10 score (*r* = 0.60; 95% CI 0.31–1.00; *P* = 0.001) and the overall liaison measure (*r* = 0.46; 95% CI 0.13–1.00; *P* = 0.013). There was no significant correlation between the CORE-10 and any of the other subscales: psychosis (*r* = 0.31; 95% CI −0.04 to 1.00; *P* = 0.072); cognition (*r* = −0.15; 95% CI −0.48 to 1.00; *P* = 0.705); substance misuse (*r* = 0.10; 95% CI −0.26 to 1.00; *P* = 0.322); mind–body (*r* = 0.24; 95% CI −0.13 to 1.00; *P* = 0.140); and behaviour (*r* = −0.06; 95% CI −0.40 to 1.00; *P* = 0.603).

### CAM

CAM scores were available for 41 patients; 11 of these were positive scores. Patients who scored positively on the CAM had a significantly higher score on the cognition subscale of the measure than those who did not (mean 7.18, s.d. 3.42 *v.* mean 0.47, s.d. 1.43; *P* < 0.001). They also had higher scores on the psychosis subscale (mean 7.37, s.d. 3.26 *v.* mean 1.50, s.d. 2.56; *P* < 0.001) and the behaviour subscale (mean 5.64, s.d. 1.51 *v.* mean 0.73, s.d. 1.68; *P* < 0.001), but not on the mood subscale (mean 1.91, s.d. 2.34 *v.* mean 2.1, s.d. 3.00; *P* = 0.612) or the substance misuse subscale (mean 3.09, s.d. 4.11 *v.* mean 1.43, s.d. 2.22; *P* = 0.441). Comparisons were made using the Mann–Whitney *U*-test for independent samples. Data for the mind–body subscale were not analysed owing to the poor interrater agreement for these items.

## Discussion

This study represents a preliminary attempt to develop an outcome measure for local use in Greater Manchester liaison psychiatry services. The findings are encouraging, but cannot currently be generalised beyond the settings involved in the evaluation. Strengths of the measure include: extensive involvement of liaison clinicians in all stages of development, particularly item generation; field testing and refinement of the measure; positive feedback from clinicians regarding ease of use and acceptability; good interrater reliability for most items and subscales, with the exception of the mind–body subscale; preliminary evidence of the instrument's stability and sensitivity to change, and reasonable comparability for two of the measure's subscales with recognised instruments used routinely for sub-populations of patients seen by liaison services.

The measure shares some similarities with HoNOS, although many items are more specific to liaison settings (items 2, 12, 13, 14, 15, 16, 17, 21 and 22). Like HoNOS, however, the measure was designed to cover a broad clinical area, rather than a specific psychological dimension.

The measure appears to have face validity in that it covers areas relevant to liaison psychiatry, and all the items were generated by working clinicians in the field. On average, it takes approximately 2 min to complete, but clinicians who are familiar with the instrument can complete it in shorter periods of time.

The heterogeneity of the instrument makes it challenging to validate in a conventional way, as each of the six subscales would need comparison with a separate recognised instrument. We compared it with two measures that are used routinely in our services. There was a significant association between the CORE-10 (a measure of psychological symptoms) and the mood subscale of the liaison instrument, which provides some support for the utility of this subscale. The cognition subscale scores correlated well with positive CAM scores, as did the psychosis and behaviour subscales. These findings provide support for the clinical utility of the instrument, as one might expect that patients who are confused and suffering from delirium may also have symptoms related to behavioural disturbance and psychosis.

It was beyond the scope of this project to use any other recognised appropriate measures for comparison with the other subscales, as no other measures are used routinely in the clinical services involved in this evaluation.

The mind–body items showed disappointingly poor interrater reliability. In the development of the scale, clinicians felt it was important to include mind–body items, but judgements as to whether behaviour or treatment-seeking are ‘disproportionate’ are difficult to make in practice. These items have subsequently been revised and rewritten for further evaluation.

Our clinician panels recommended inclusion of contextual items in addition to the main measure, in order to assess the complexity of patients’ physical, mental and social status. We will report in detail on the utility of these additional baseline items in a subsequent report.

The measure was primarily tested on acute general hospital wards; we are currently exploring the potential utility of the measure in out-patient and emergency department settings with a view to field testing. In addition, most of the raters were doctors, as opposed to nurses. This reflected the staffing of the two services involved in the evaluation, and the requirement of psychiatric trainees to have training in audit and service evaluation. The measure has no items that require specific medical expertise; further evaluation of its use by nursing staff would be informative.

The main purpose of developing the measure was to be able to record symptomatic outcomes in our local services, which would be credible and informative. Despite the above caveats, we believe the measure is better than any other currently available instrument for recording overall outcomes in the liaison setting, and it has been adopted locally and incorporated into an electronic format for routine use, in addition to the FROM-LP framework.

The measure requires further development and field testing in different settings before it can be recommended for widespread use. With this in mind, we are now in the process of applying for funding and ethical approval for a more robust evaluation of the instrument.

## About the authors

**Elspeth Guthrie** is a professor of psychological medicine at the University of Leeds. **Mathew Harrison** is a specialist registrar in general adult psychiatry and medical psychotherapy in the Leeds and York Partnership NHS Foundation Trust. **Richard Brown** is a senior lecturer in clinical psychology and honorary consultant clinical psychologist in the Division of Psychology and Mental Health, School of Health Sciences, University of Manchester, Manchester Academic Health Sciences Centre. **Rajdeep Sandhu** is a locum consultant in old age psychiatry in the Pennine Care NHS Foundation Trust. **Peter Trigwell** is a consultant in psychological medicine in the Leeds and York Partnership NHS Foundation Trust. **Seri Abraham** and **Shazada Nawaz** are specialist registrars in general adult psychiatry in the Lancashire Care NHS Foundation Trust. **Peter Kelsall** is a specialist registrar in general adult psychiatry in the Pennine Care NHS Foundation Trust. **Rachel Thomasson** is a consultant neuropsychiatrist in the Salford Royal NHS Foundation Trust.
